# Global Burden of Headache Disorders in Children and Adolescents 2007–2017

**DOI:** 10.3390/ijerph18010250

**Published:** 2020-12-31

**Authors:** Matilde Leonardi, Licia Grazzi, Domenico D’Amico, Paolo Martelletti, Erika Guastafierro, Claudia Toppo, Alberto Raggi

**Affiliations:** 1 UOC Neurologia, Salute Pubblica, Disabilità, Fondazione IRCCS Istituto Neurologico Carlo Besta, 20133 Milan, Italy; erika.guastafierro@istituto-besta.it (E.G.); claudia.toppo@istituto-besta.it (C.T.); alberto.raggi@istituto-besta.it (A.R.); 2 UOC Neuroalgologia, Fondazione IRCCS Istituto Neurologico Carlo Besta, 20133 Milan, Italy; licia.grazzi@istituto-besta.it (L.G.); domenico.damico@istituto-besta.it (D.D.); 3 Department of Clinical and Molecular Medicine, Sapienza University, 00185 Rome, Italy; paolo.martelletti@uniroma1.it

**Keywords:** headaches, migraine, tension-type headache, TTH, prevalence, Years Lived with Disability, YLDs, Global Burden of Disease, children, adolescents

## Abstract

Headache disorders are prevalent and disabling conditions impacting on people of all ages, including children and adolescents with substantial impact on their school activities and leisure time. Our study aims to report specific information on headaches in children and adolescents based on the Global Burden of Disease (GBD) study, that provides estimates for incidence, prevalence, fatal and non-fatal outcomes. We relied on 2007 and 2017 GBD estimates for prevalence and Years Lived with Disability (YLDs) at the global level and in WHO regions. The results show that, migraine and tension-type headache (TTH) together account for 37.5% of all-cause prevalence and for 7% of all-cause YLDs. Over the past decade, prevalence rates showed a mild increase of TTH in all ages and of migraine alone for adolescents. The YLDs increased among females of all ages with some regional differences that might be connected to the unequal availability of effective acute and prophylactic treatments across world regions. GBD data support the need to promote public health policies and strategies including diagnosis, pharmacological and non-pharmacological treatments that are expected to help reduce the disability and burden associated to migraine and TTH among children and adolescents.

## 1. Introduction

Primary headache disorders affect people of all ages but usually peak in adult populations. The way a child exhibits a headache may be related to many factors, such as genetics, hormones, stress, diet, medications, and dehydration. Recurrent headaches of any type can cause school problems, behavioral problems, and/or depression. Literature addressing prevalence of primary headache disorders among children and adolescent exists, in particular, two literature review found overall prevalence of primary headaches at 58.4% [1] and 54.4% [2] of children and adolescents, with migraine alone affecting 7.7% (95% CI 7.6–7.8%) [1] and 9.1% (95% CI 7.1–11.1%) [2], with a clear gender gradient. Although large population studies on the very young, below six years of age are basically lacking, in pediatric populations migraine and other primary headaches substantially impact on health, on school-related activities, on participation in family, sport and social activities, as well as on quality of life [3,4,5,6]. Treatment opportunities are quite limited, especially specific pharmacological approaches [6], whereas non-pharmacological ones, such as behavioral therapies, are deemed to produce a good therapeutic effects. [7,8]. Also, patients’ and parents’ education on the importance of life-style issues are important [7].

The Global Burden of Disease (GBD) study produces estimates for incidence, prevalence, fatal and non-fatal outcomes, offering an approach to health loss quantifying and enabling comparisons over time, across conditions, countries and world region. Primary headache disorders have received large public health attention for the last 20 years, also due to the “Lifting the Burden” campaign [9,10], which was able to raise attention and awareness on the issue of the burden of headache disorders. However, such a level of attention was mostly directed to the adult population and to indicators of productivity loss [11]. The estimates of the GBD 2016 wave showed that migraine is the first cause of disability in people aged 15–49, and it ranked second in all-causes list, being responsible for 5.6% of all YLDs (Years Lived with Disability) in the world [12]. In addition to this, a recent review addressing the “operational content” of burden of migraine, which is the most burdensome primary headache, showed that “migraine burden” is underlined by broad concepts that are poorly suitable for populations of children and adolescents [13].

Large-scale epidemiological studies on children and adolescents in which primary headache disorders are included and vice-versa are lacking. The GBD Study represents a valid approach to present global and regional estimates on headaches prevalence and disability. In this study, we relied on the 2007 and 2017 waves of the GBD study to present such information, at global and at regional levels, considering the six WHO regions, as well as the variation over the decade.

## 2. Materials and Methods

We used the GBD 2017 and 2007 estimates made available by the Institute for Health Metrics and Evaluation (available at http://ghdx.healthdata.org/gbd-results-tool last access: 15/10/2020) referred to tension-type headache (TTH) and migraine for children and adolescents (i.e., age group 5–9, 10–14 and 15–19): Younger age groups are not included as GBD estimates for headache are not calculated for people aged less than 5. The estimates are referred to migraine and TTH as a whole, with no distinction between episodic and chronic forms and, for migraine, between migraine with and without aura.

We used global estimates and estimates for each of the six WHO regions, i.e. African Region, Eastern Mediterranean Region, European Region, Region of the Americas, South-East Asia Region and Western Pacific Region.

The estimates were descriptively reported; at global level estimates were reported in numbers and rates/100,000, whereas at WHO regions levels estimates were reported as rates/100,000 only. We reported estimates for migraine and TTH prevalence and YLDs, by age group (5–9, 10–14, 15–19 years of age), gender and by WHO region. We reported estimates for years for 2007 and 2017, and for the variation 2007–2017. For each estimate we reported the mean value and the 95% Uncertainty Intervals (95%UI). No direct mortality is associated to headache disorders and Years of Life Lost (YLLs) are not computed. Thus, Disability-Adjusted Life Years (DALYs), composed of YLLs and YLDs—correspond to YLDs.

## 3. Results

At the global level, the population of people aged 5–19 was 1.86 billion in 2007, mostly living in South-East Asia Region (29.6%) and Western Pacific Region (21.4%), whereas in 2017 the corresponding figures were 1.91 billion people mostly living in South-East Asia Region (29.7%) and African Region (20.4%); in both years, females represented 48.5% of the entire population. The population growth in the period was 2.7%.

### 3.1. Migraine and TTH Prevalence Estimates

At global level, there were 698.2 million people aged 5–19 with migraine or TTH in 2007 and 716.8 in 2017, with an increase comprised between 0.7% (the younger groups) and 1.6% (the 15–19 age group). The two conditions together represent approximately 37.5% of all-cause prevalence in both years, with TTH accounting for around 72% of all cases, and migraine the remaining 28%.

**Figure 1:**
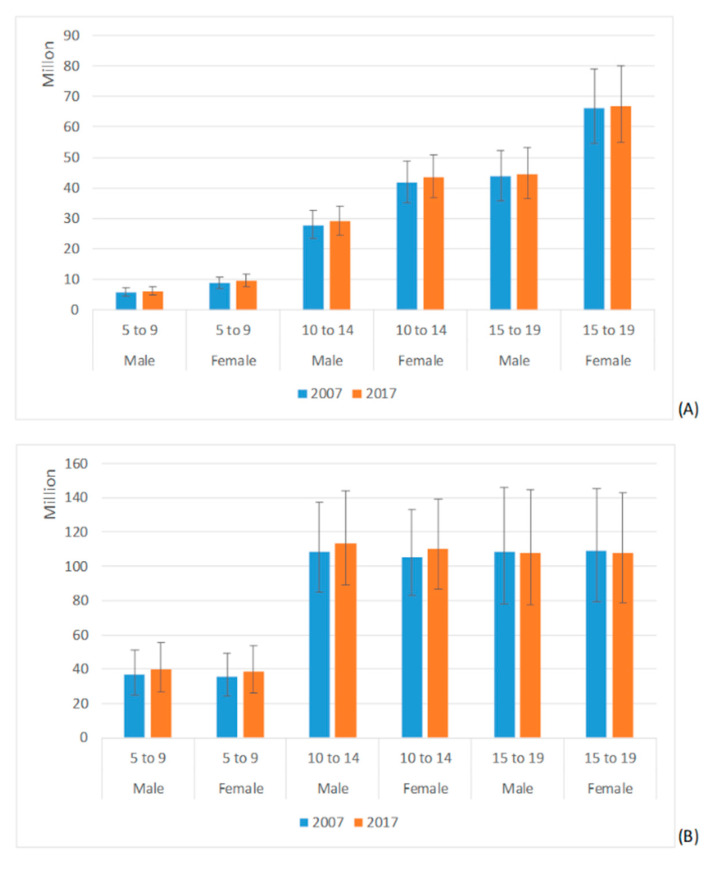
Global prevalence of migraine, and (**A**) TTH (**B**) by gender and age group. Estimates are reported in millions, with mean and 95% UI.

Figure 1 shows the prevalence of migraine and TTH at global level. For migraine, a clear gender and age gradient exists, with females and older subjects showing higher prevalence, whereas for TTH a clear difference existed between the younger group and the two older ones.

Table 1 shows prevalence rates for each age group, gender and world region in the two years, as well as changes over the decade. At the global level a significant increase in prevalence rates was observed: for migraine, it was limited to the older group, by 3.4% among females and by 3.8% among males, whereas for TTH a 0.5–1.3% increase in all age groups was observed.

In all Regions, migraine prevalence rates were generally stable between 2007 and 2017 with some exceptions for the older group: An increase in the African in the European and in the Western Pacific regions, and a decrease in the Eastern Mediterranean Region. TTH prevalence rates showed an increase in all age groups in the African and in South-East Asia regions, in the 10–14 and 15–19 age group in the Eastern Mediterranean Region, in the 10–14 age group in the Region of the Americas; conversely, rates decreased in the older age group in the European Region. All the remaining groups and regions showed stable prevalence rates.

### 3.2. Migraine and TTH YLDs Estimates

At global level, there were 114.6 million YLDs in 2007 and 114.9 in 2017: Migraine and TTH together accounted for 8.0 and 8.2 million YLDs (i.e., around 7% of all-cause YLDs, around 11% of all NCDs-associated YLDs and around 72% of all YLDs associated to neurological disorders). Between 2007 and 2017, YLDs associated to headache disorders increased with a percentage comprised between 1.8% (age group 10–14, not significant) and 6.5% (age group 5–9).

**Figure 2:**
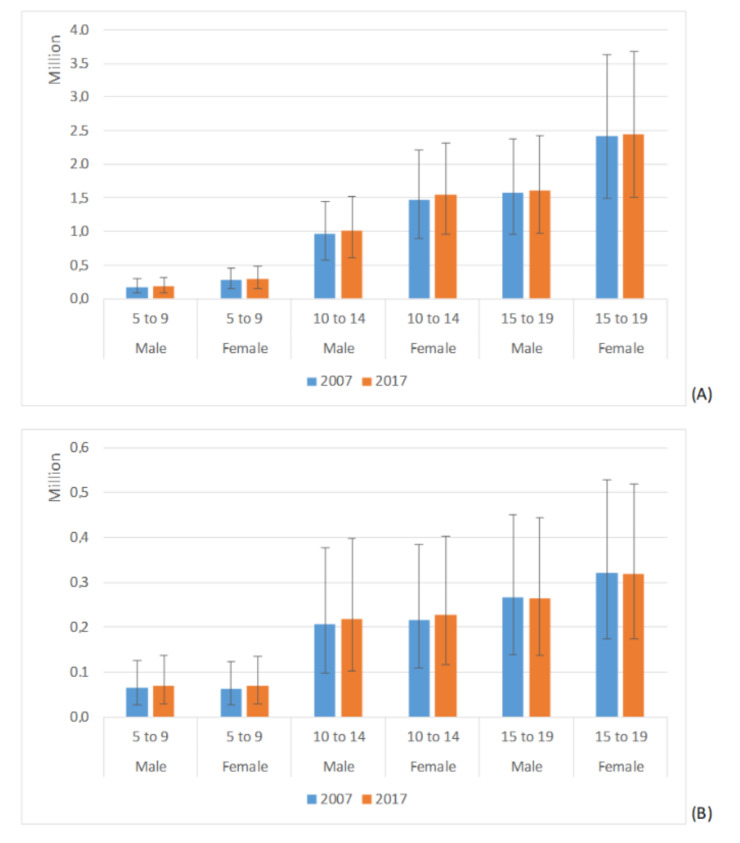
YLDs associated to migraine (**A**) and TTH (**B**) by gender and age group at global level. Estimates are reported in millions, with mean and 95% UI.

Figure 2 shows the YLDs associated to migraine and TTH at global level. For migraine, a clear gender and age gradient exists, with females and older subjects showing higher YLDs, whereas for TTH a difference exists between the younger group and the two older ones.

Table 2 shows YLDs rates for each age group, gender, world region in the two years, as well as percentage change. Over the 2007–2017 period, at the global level a significant increase in YLDs rates change was observed: for migraine, it was limited to the older group, by 4.4% among females and by 2.7% among males, whereas for TTH the increase was observed mostly among females in all age groups, by 2.5–6.6%, and among males only in the younger group by 7.8%.

In the single regions, with regard to migraine, YLDs rates were generally stable between 2007 and 2017 with some exceptions: They increased in the African Region, but only for females in the older group, and in the South-East Asia Region, limitedly to children aged 5–9 of both sexes, and adolescent females aged 15–19. With regard to TTH, YLDs rates increased in females in all age groups, and for males in the younger group in the South-East Asia Region. All the remaining groups and regions showed stable YLDs rates.

## 4. Discussion

With this paper we present the global estimates for prevalence and YLDs referred to TTH and migraine among children and adolescents. At a global level, our results indicate that in the decade 2007–2017 prevalence rates showed a mild increase in all ages for TTH and among adolescents for migraine. YLD rates also showed some increase, mostly referred to the female population. The observed increase in prevalence and in YLDs rates are however not uniform, and regional differences exist. Globally in both 2007 and 2017, migraine and TTH accounted for 37.5% of all-cause prevalence and for 7% of all-cause YLDs.

With regard to prevalence rates, migraine showed an increase mostly in African countries and, among adolescents only, in European and Western Pacific countries. With regard to TTH, the increase in prevalence rates was evident in the African, Eastern-Mediterranean and South-East Asia regions.

Compared to the changes in prevalence rates, the increase observed in YLDs was larger particularly among females and with particular reference to TTH-related disability. Such a change is likely due to the dramatic increase in YLDs rates observed in South-East Asia Region.

The information presented here is based upon GBD estimates, a data modelling based on real world data, including studies and population censuses. For example, GBD 2016 estimates on migraine were extracted from 176 studies, and estimates on TTH from 126 studies. Clearly, not all countries or sub regions are covered, and most of studies are from western Europe, high-income North America, and high-income Asia Pacific regions. In all regions and countries, prevalence was estimated with a Bayesian meta-regression model (DisMod-MR 2.1), and estimates were obtained in this way also for countries and regions where no relevant headache studies had been done. Such an approach is of clear added value in the case of the estimates herein presented, as primary data on populations of children and adolescents are scarce. Therefore, GBD estimates represent, for many regions, the unique source of information for health policy planning and provision of effective medications or other treatments.

Globally, as stated in the *Lancet 2020 Campaign on child and adolescents* the economic, political, commercial, and environmental determinants, so essential for child and adolescent health are changing dramatically. Much positive changes have happened in child and adolescent health in recent years but children and youth growing up today face new and unprecedented threats to their health [14]. The COVID-19 pandemic has exacerbated many of these threats, jeopardizing child welfare gains and causing a global economic and health crisis, which will impact children and adolescents in terms of economic disadvantage, health and access to services [15,16].

Our paper shows that over a decade, despite the improvements in diagnosis and treatments of primary headaches in all ages, a minor impact, or no impact at all, on disability associated to these disorders was shown at global level, also making reference to GBD 2016 estimates [11]. These years in fact have seen the publication of some studies addressing the safety and efficacy of triptans also among adolescents [17,18,19], providing a possible alternative to approaches based on paracetamol and ibuprofen. Concerning primary headache prevention there are available drugs, used in adults, that can be prescribed to children and adolescents as well [20,21]. Although, specific attention is needed in pediatric population in consideration of specific risks and side effects, such as hormonal interactions, sleepiness and cognitive slowing [8]. The reasons for the lack of improvement in YLD rates might also be due to the unequal availability of drugs, including headache-specific therapies across the world, as well as the increased prevalence of headache disorders in reason of higher diagnostic ability.

It is now increasingly clear that in the treatment of primary headaches of children and adolescents, non-pharmacological approaches are also to be considered. As shown in a recent review, such treatments produced sizeable effects on headache frequency, with a 34–78% reduction, which is similar to that observed in trials on pharmacological treatments [22,23]. Based on the considerations reported above, non-pharmacological treatments could be proposed together with pharmacological ones. Such a combined approach might be of benefit for a wide set of the pediatric population and might help considerably reducing headache frequency, and thus, limit the impact on school and leisure time activities. The impact of migraine on a child’s quality of life and disability has been well-documented, being emotional issues and school performance among the most common problems [3,4,5,6].

High-quality population data on headache disorders among children and adolescents are largely lacking. The most outstanding example of data collection referred to this specific population is the global schools-based program within the Global Campaign against Headache [24,25,26,27,28], which encompasses a specific approach and a set of instruments to address prevalence and impact of primary headache disorders in children (aged 6–11) and adolescents (aged 12–17) [29]. More studies are needed in different countries and regions to enhance the estimates produced by future GBD iterations and to raise global awareness of a burden that can and should be reduced so as to decrease global children and adolescents’ disability worldwide.

### Limitations

This study suffers from the general limitations of all GBD studies. First, the adjustments of non-fatal outcomes to account for biases introduced by different case definitions, e.g., registries based on medical data versus patients’ reports. This is particularly critical for the estimates presented here by reason of the lack of large studies specifically addressing headaches in populations of children and adolescents. Second, the 95% UIs used to define the precision of the estimates are sometimes wide, reflecting the overall uncertainty of the estimates: this is particularly true for TTH prevalence estimates. Third, the adjustment for comorbidities, which is made with the assumption of the independent distribution of comorbid conditions is critical considering that headache disorders are comorbid to a large amount of conditions and vice versa. Fourth, the estimates herein presented are not complete as we decided to focus on TTH and migraine (fourth-level conditions) and not on the third-level “Headache Disorders”. The result of this is that some headache disorders that were not included in the fourth level are not considered in this analysis. The most common condition among the entire span of age groups is cluster headache, which is a rare condition in the general all-age population, and even rarer among children and adolescents.

## 5. Conclusions

In conclusion, we report global and regional estimates of migraine and TTH prevalence and YLDs in population of children and adolescents. The estimates presented here show that, in both 2007 and 2017, migraine and TTH accounted for 37.5% of all-cause prevalence and for 7% of all-cause YLDs at the global level, with a mild increase in prevalence and YLDs, the latter mostly referred to the female groups.

Such an increase in YLDs and YLD rates might be connected to the unequal availability of access to healthcare facilities for prophylaxis, diagnosis, as well as treatment across different world regions. Action is needed to promote care at primary, secondary and tertiary level, complementing pharmacological and non-pharmacological treatments that are expected to help reduce the disability associated to primary headache disorders among children and adolescents.

## Figures and Tables

**Table 1:** Prevalence rates/100,000 for 2007 and 2017 and percentage variation, for migraine and TTH, by age and gender, at global level and by WHO regions.

Location and Age Range	Females			Males		
	2007	2017	Variation	2007	2017	Variation
**Global-Migraine**						
5–9	2996.0 (2387.9;3652.4)	2956.3 (2350.1;3613.2)	−0.7% (−4.2;2.8%)	1809.3 (1431.6;2237.5)	1795.6 (1417.7;2211.8)	0.1% (−3.7;3.7%)
10–14	14,149.7 (11,954.0;16,563.3)	14,144.4 (11,902.9;16,581.7)	0.1% (−2.4;2.9%)	8803.0 (7432.0;10381.5)	8832.4 (7426.5;10,389.0)	0.7% (−2.1;3.3%)
15–19	21,529.3 (17,769.9;25,636.3)	22,279.6 (18,332.4;26,664.4)	3.4% * **(1.5;5.4%)**	13,596.9 (11,093.5;16,178.2)	14,121.7 (11,514.5;16,906.4)	3.8% * **(1.7%;5.9%)**
**Global-TTH**						
5–9	12,126.6 (8189.7;16,770.1)	12,142.7 (8195.7;16,807.5)	0.7% * **(0.3;1.2%)**	11,657.6 (7812.0;16,193.8)	11,668.6 (7796.9;16,222.8)	0.9% * **(0.4;1.5%)**
10–14	35,624.1 (28,047.0;45,105.4)	35,748.8 (28,145.9;45,226.8)	0.5% * **(0.1;0.9%)**	34,243.9 (26,829.1;43,514.0)	34,466.6 (27,033.1;43,781.5)	1.0% * **(0.6;1.4%)**
15–19	35,451.0 (25,841.4;47,154.6)	35,834.4 (26,156.8;47,624.5)	1.0% * **(0.3;1.7%)**	33,570.2 (24,124.9;45,204.0)	34,035.6 (24,528.0;45,761.0)	1.3% * **(0.8;1.9%)**
**African Region-Migraine**						
5–9	2052.5 (1581.6;2598.0)	2081.3 (1599.8;2631.9)	2.7% * **(1.7;3.5%)**	1135.2 (1024.5;1690.8)	1351.1 (1034.6;1716.0)	2.7% * **(2.0;3.4%)**
10–14	10,465.5 (8600.3;12,687.8)	10,569.0 (8675.9;12,808.5)	2.0% * **(1.2;2.7%)**	6877.5 (5660.1;8249.4)	6909.8 (5674.3;8304.2)	1.8% * **(1.2;2.4%)**
15–19	19,155.9 (15,427.3;23,222.6)	19,166.8 (15,375.2;23,334.6)	0.4% * **(0.0;0.9%)**	12,477.2 (9923.8;15,198.0)	12,431.8 (9872.0;15,179.8)	**0.2%** **(−0.3;0.6%)**
**African Region-TTH**						
5–9	11,707.7 (7879.1;16,349.5)	11,835.3 (7981.8;16,531.3)	2.4% * **(2.0;2.8%)**	11,275.9 (7522.4;15826.3)	11,352.9 (7561.0;15,942.2)	2.1% * **(1.7;2.7%)**
10–14	34,222.3 (26,820.3;43,450.5)	34,544.8 (27,177.5;44,000.4)	1.9% * **(1.6;2.3%)**	33,317.1 (25,951.2;42,565.5)	33,520.8 (26,148.0;42,788.6)	2.0% * **(1.5;2.4%)**
15–19	3816.8 (22,911.6;43,090.1)	31,965.9 (22,998.0;43,367.8)	0.8% * **(0.5;1.2%)**	31,331.1 (22,472.0;42,730.0)	31,399.5 (22,485.1;72,857.0)	0.7% * **(0.4;1.1%)**
**Eastern Mediterranean Region-Migraine**						
5–9	3085.0 (2414.2;3832.0)	3080.9 (2413.1;3831.3)	0.9% (−0.2;1.8%)	2130.0 (1682.5;2651.9)	2128.9 (1680.5;2652.9)	1.1% (−0.2;2.3%)
10–14	14,845.1 (12,457.2;17,616.9)	14,703.6 (12,325.8;17,460.0)	0.3% (−0.5;1.0%)	10,590.8 (8885.9;12,500.6)	10,441.8 (8765.9;12,343.5)	0.0% (−0.2;2.3%)
15–19	24,453.3 (20,967.9;30,305.5)	24,618.6 (20,242.8;29,329.0)	−2.9% * **(−3.5;−2.1%)**	17,597.3 (14,219.9;21,080.4)	16,937.4 (13,713.8;20,306.4)	−3.1% * **(−3.8;−2.3%)**
**Eastern Mediterranean Region-TTH**						
5–9	12,011.3 (8129.3;16,698.2)	11,983.4 (8098.3;16,696.2)	0.8% (−0.1;1.6%)	11,866.8 (7982.3;16,648.0)	11,788.4 (7947.9;16,539.7)	0.5% (−0.8;1.7%)
10–14	33,710.3 (26,507.7;42,641.6)	33,813.4 (2660.7;42,720.7)	1.5% * **(0.9;2.2%)**	33,558.9 (26,109.2;42,987.0)	33,588.1 (26,100.7;43,024.1)	1.5% * **(0.5;2.4%)**
15–19	31,830.2 (23,357.2;42,070.9)	32,296.5 (23,751.1;42,860.9)	1.9% * **(1.1;2.8%)**	32,499.5 (23,500.6;43,162.6)	32,870.6 (23,835.9;43,546.3)	1.8% * **(1.1;2.6%)**
**European Region-Migraine**						
5–9	3207.2 (2576.9;3885.5)	3248.3 (2574.0;3989.0)	1.7% (−1.4;4.8%)	1786.5 (1424.3;2171.7)	1801.4 (1435.8;2202.1)	1.4% (−1.3;4.1%)
10–14	15,289.1 (12,948.;17,765.2)	15,510.5 (13,014.0;18,195.6)	−0.1% (−2.4;2.9%)	8745.4 (7377.4;10,258.2)	8835.6 (7383.6;10,451.9)	2.1% (−0.1;4.2%)
15–19	25,126.0 (20,934.7;29,465.5)	25,518.8 (21,077.1;30,274.2)	1.9% * **(0.0;3.7%)**	14,197.9 (11,670.8;16,884.9)	14,367.2 (11,747.6;17,158.9)	2.1% * **(0.4;3.9%)**
**European Region-TTH**						
5–9	11,302.7 (7712.5;15706.4)	11,458.8 (7673.8;16,003.2)	1.7% (−0.6;3.6%)	12,246.9 (8348.3;16,937.5)	12,347.6 (8380.1;17,218.8)	1.4% (−0.7;3.3%)
10–14	33,272.1 (26,430.7;41,961.4)	32,936.1 (26,060.6;41,486.1)	−0.1% (−1.6;1.2%)	34,875.9 (27,502.8;44,034.4)	34,777.0 (27,361.1;44,006.5)	0.7% (−0.7;1.9%)
15–19	33,290.1 (24,364.4;44,377.1)	32,010.0 (23,390.6;42,898.1)	−3.5% * **(−4.9;−2.2%)**	32,659.7 (23,684.4;43,390.8)	31,838.9 (22,949.6;42,334.4)	−1.7% * **(−2.9;−0.4%)**
**Region of the Americas-Migraine**						
5–9	4762.4 (3818.2;5781.2)	4772.7 (3836.7;5772.3)	1.3% (−2.5;4.5%)	2608.5 (2107.2;3165.7)	2574.2 (2061.3;3145.7)	−0.2% (−4.5;3.7%)
10–14	21,409.6 (18,335.4;24,836.1)	21,401.3 (18,331.4;24,949.7)	0.9% (−1.8;3.4%)	12,024.3 (10,238.7;14,012.9)	11,908.0 (10,092.2;13,899.9)	0.3% (−2.7;3.4%)
15–19	30,015.5 (25,058.5;35,166.5)	30,089.5 (25,096.2;35,441.1)	0.5% (−1.5;2.7%)	16,563.6 (13,719.9;19,601.1)	16,625.5 (13,844.3;19,611.4)	0.9% (−1.7;3.3%)
**Region of the Americas-TTH**						
5–9	11,961.3 (8214.4;16,403.7)	11,954.7 (8189.1;16,544.1)	1.0% (−0.9;3.0%)	12,090.7 (8246.2;16,704.3)	12,042.8 (8164.2;16,625.3)	0.8% (−2.1;2.9%)
10–14	34,347.7 (26,851.8;43,588.6)	34,452.8 (26,929.6;43,882.1)	1.3% * **(0.0;2.7%)**	34,247.8 (26,567.2;43,930.0)	34,606.7 (26,881.8;44,340.7)	2.4% * **(0.9;4.0%)**
15–19	34,662.6 (25,118.6;46,253.2)	34,419.2 (25,001.3;45,595.6)	−0.4% (−1.6;0.9%)	35,102.0 (25,581.8;46,030.0)	34,451.5 (25,051.3;45,221.5)	−1.4% (−2.8;0.1%)
**South-East Asia Region-Migraine**						
5–9	3428.3 (2741.3;4168.3)	3404.0 (2692.8;4178.2)	0.2% (−10.0;10.6%)	2072.2 (1626.3;2563.1)	2072.1 (1647.4;2554.3)	1.5% (−8.4;12.7%)
10–14	16,007.0 (13,553.9;18,700.3)	15,964.8 (13,453.1;18,645.7)	0.2% (−8.1;8.8%)	10,163.5 (8555.1;12,078.3)	10,163.9 (8551.3;12,033.6)	1.2% (−6.3;9.0%)
15–19	23,648.0 (19,474.7;28,274.5)	23,818.6 (19,588.0;28,437.9)	0.9% (−4.6;6.9%)	16,087.6 (13,075.2;19,174.9)	16,090.1 (13,091.5;19,337.4)	0.6% (−4.7;6.2%)
**South-East Asia Region-TTH**						
5–9	13,397.7 (8927.3;18,707.9)	13,407.3 (8914.8;18,716.6)	1.0% * **(0.6;1.4%)**	12,674.7 (8499.1;17,667.7)	12,665.5 (8485.3;17,675.9)	1.4% * **(0.8;2.0%)**
10–14	40,273.4 (31,690.9;50,486.9)	40,317.5 (31,756.6;50,534.9)	0.6% * **(0.3;0.8%)**	38,162.0 (29,879.8;48,013.5)	38,179.6 (29,908.0;48,013.4)	1.2% * **(0.8;1.8%)**
15–19	43,507.2 (32,092.3;56,830.4)	43,734.6 (32,274.1;57,079.0)	0.7% * **(0.5;0.9%)**	39,754.9 (28,736.1;53,602.0)	39,820.7 (28,782.9;53,716.4)	**0.8%** **(0.5;1.1%)**
**Western Pacific Region-Migraine**						
5–9	1825.0 (1458.7;2241.1)	1824.6 (1453.7;2244.7)	0.0% (−1.4;1.2%)	1132.7 (871.1;1406.6)	1142.3 (878.6;1416.6)	0.8% (−0.1;2.0%)
10–14	8844.8 (7409.2;10,461.2)	8935.5 (7482.7;10,579.6)	0.5% (−0.6;1.4%)	5507.5 (4579.2;6552.6)	5598.1 (4656.5;6660.3)	0.7% (−0.2;1.7%)
15–19	13,612.1 (11,073.6;16,269.0)	14,313.9 (11,667.1;17,144.7)	5.1% * **(3.9;6.2%)**	8393.0 (6850.8;10,031.9)	9034.6 (7349.3;10,800.6)	7.4% * **(6.3;8.4%)**
**Western Pacific Region-TTH**						
5–9	10,944.1 (7280.5;15,354.0)	10,975.3 (7304.4;15,401.6)	0.3% (−0.3;1.0%)	9708.5 (6460.3;13,684.9)	9754.0 (6533.6;13,755.5)	0.5% (−0.3;1.3%)
10–14	32,870.7 (25,391.7;42,431.6)	32,868.7 (25,484.6;42,417.7)	−0.5% (−1.1;0.0%)	29,497.2 (22,606.2;37,697.9)	29,653.3 (22,730.1;37,867.9)	−0.4% (−1.2;0.3%)
15–19	31,591.7 (22,560.1;43,096.8)	31,418.4 (22,458.1;43,017.8)	−0.6% (−1.5;0.3%)	28,113.1 (19,748.1;39,524.4)	28,480.9 (20,077.5;39,700.1)	1.1% * **(0.1;2.1%)**

**Note**. Percentages in bold and with * indicate significant variation.

**Table 2:** YLDs rates/100,000 for 2007 and 2017 and percentage variation, for migraine and TTH, by age and gender, at global level and by WHO regions.

Location and Age Range	Females			Males		
	2007	2017	Variation	2007	2017	Variation
**Global-Migraine**						
5–9	91.2 (47.5;152.9)	90.5 (47.3;150.9)	5.2% (−1.1;11.5%)	53.3 (25.8;93.3)	53.2 (25.6;93.3)	7.3% (−0.6;16.4%)
10–14	499.9 (305.7;748.9)	502.0 (309.3;751.3)	2.1% (−1.0;5.5%)	305.2 (182.8;457.3)	307.3 (182.5;463.6)	1.4% (−2.5;5.5%)
15–19	787.1 (483.0;1178.7)	817.0 (501.4;1227.1)	4.4% * **(2.1;7.0%)**	489.0 (294.1;737.5)	507.6 (307.4;768.1)	2.7% * **(0.3;5.6%)**
**Global-TTH**						
5–9	21.3 (9.0;41.8)	21.4 (8.9;42.2)	6.6% * **(2.6;10.8%)**	20.0 (8.2;39.6)	20.1 (8.2;8.2;39.7)	7.8% * **(2.3;13.5%)**
10–14	73.4 (37.0;130.4)	74.0 (37.6;130.8)	2.6% * **(0.0;5.5%)**	65.4 (30.9;119.1)	66.0 (31.3;121.0)	1.7% (−1.8;5.0%)
15–19	104 (56.0;171.3)	105.9 (57.4;173.3)	2.5% * **(0.3;4.9%)**	82.6 (42.7;139.3)	83.7 (42.9;140.4)	0.3% (−2.1;2.5%)
**African Region-Migraine**						
5–9	62.6 (31.8;106.1)	63.6 (32.1;107.5)	7.0% (−7.1;24.9%)	39.4 (18.7;69.2)	40.0 (18.7;69.7)	8.8% (−11.7;31.6%)
10–14	381.4 (235.0;567.0)	386.5 (239.0;583.0)	5.8% (−1.5;13.7%)	246.9 (150.8;368.0)	248.5 (149.2;368.6)	5.1% (−4.4;16.0%)
15–19	736.3 (452.7;1114.0)	739.4 (458.1;1116.3)	5.2% * **(0.2;10.7%)**	474.4 (290.1;706.8)	474.3 (286.5;714.2)	3.7% (−3.0;10.1%)
**African Region-TTH**						
5–9	20.7 (8.8;40.4)	20.9 (8.8;41.1)	6.7% (−2.5;16.7%)	19.4 (8.1;38.4)	19.6 (7.9;38.8)	8.2% (−5.5;21.1%)
10–14	73.9 (38.5;128.5)	74.8 (38.5;130.6)	5.7% (−2.1;13.8%)	65.8 (32.6;117.4)	66.2 (32.4;117.7)	5.1% (−4.2;14.7%)
15–19	109.5 (60.0;179.1)	110.0 (59.8;179.0)	5.3% (−1.3;12.0%)	86.1 (45.1;143.4)	86.2 (45.3;143.3)	3.9% (−3.3;11.2%)
**Eastern Mediterranean Region-Migraine**						
5–9	97.2 (53.4;159.3)	97.4 (53.0;160.3)	5.5% (−11.7;25.7%)	66.5 (35.1;111.4)	66.8(34.8;110.8)	2.4% (−19.5;28.3%)
10–14	543.6 (335.0;806.4)	539.1 (329.4;811.1)	1.2% (−6.1;8.8%)	385.4 (232.5;576.2)	379.5 (228.8;562.3)	−1.5% (−19.5;28.3%)
15–19	953.2 (585.2;1418.7)	918.8 (562.4;1379.8)	−1.5% (−6.8;4.3%)	661.2 (399.9;995.5)	632.1 (385.8;953.7)	3.6% (−8.5;1.9%)
**Eastern Mediterranean Region-TTH**						
5–9	21.3 (9.1;42.2)	21.3 (9.3;41.4)	5.4% (−8.8;20.0%)	20.6 (8.1;40.0)	20.4 (8.1;39.7)	1.3% (−8.6;23.3%)
10–14	73.1 (38.0;128.5)	73.2 (37.7;128.0)	2.5% (−5.8;11.8%)	66.2 (31.6;120.0)	66.2 (31.9;116.7)	0.0% (−11.6;11.7%)
15–19	111.5 (60.2;180.8)	19.6 (59.7;178.8)	0.5% (−7.7;8.5%)	88.8 (47.2;145.6)	87.4 (45.6;146.3)	−0.8% (−8.8;7.3%)
**European Region-Migraine**						
5–9	100.2 (53.5;165.6)	101.7 (54.0;168.2)	2.4% (−5.5;10.4%)	54.3 (26.7;95.0)	54.5 (27.0;93.5)	2.5% (−9.5;15.8%)
10–14	559.4 (342.7;828.1)	568.0 (350.3;846.2)	2.6% (−2.0;6.8%)	309.7 (186.6;469.2)	312.6 (187.9;471.5)	1.7% (−3.2;6.9%)
15–19	968.2 (602.7;1446.4)	979.9 (610.0;1476.2)	0.7% (−2.2;3.8%)	518.5 (317.4;782.9)	522.0 (315.5;791.4)	1.2% (−2.7;4.9%)
**European Region-TTH**						
5–9	20.4 (8.7;39.5)	20.6 (8.7;40.3)	2.3% (−3.9;8.5%)	21.2 (8.6;41.4)	21.4 (8.7;42.4)	2.9% (−3.9;11.0%)
10–14	76.9 (40.6;131.6)	76.7 (40.0;131.9)	0.8% (−3.4;5.7%)	68.6 (33.5;122.8)	68.5 (33.0;123.6)	0.7% (−4.0;5.5%)
15–19	127.4 (70.4;204.9)	123.6 (67.7;199.1)	−3.4% (−7.2;0.6%)	88.2 (46.8145.9)	85.5 (44.7;140.3)	−2.5% (−6.5;1.5%)
**Region of the Americas-Migraine**						
5–9	150.1 (83.0;243.2)	150.7 (83.0;245.0)	0.3% (−6.4;7.6%)	79.2 (40.0;133.4)	78.1 (39.4;132.0)	−0.9% (−9.9;9.2%)
10–14	762.1 (465.2;1140.3)	726.7 (470.0;1139.2)	−1.1% (−4.9;3.4%)	417.5 (254.6;628.6)	413.8 (250.0;624.2)	−1.4% (−6.3;4.0%)
15–19	1070.4 (651.2;1604.7)	1075.9 (654.0;1600.4)	2.1% (−1.3;5.7%)	575.0 (345.0;873.2)	579.0 (350.4;878.3)	1.6% (−2.2;5.8%)
**Region of the Americas-TTH**						
5–9	20.9 (8.7;41.2)	20.9 (8.7;41.5)	0.1% (−6.0;6.0%)	20.7 (8.5;41.4)	20.6 (8.3;40.9)	0.2% (−6.7;7.4%)
10–14	69.5 (34.1;124.2)	69.8 (34.1;124.0)	−0.7% (−4.8;3.3%)	64.0 (29.6;116.9)	64.7 (29.8;117.2)	0.5% (−3.9;5.4%)
15–19	96.8 (51.3;160.1)	96.5 (51.4;159.9)	1.3% (−2.7;5.0%)	80.7 (40.6;136.8)	79.8 (40.4;135.3)	−0.2% (−3.9;3.8%)
**South-East Asia Region-Migraine**						
5–9	102.1 (51.3;172.9)	102.1 (52.9;178.2)	17.6% * **(3.1;34.3%)**	58.6 (26.3;109.4)	59.3 (26.9;108.1)	23.0% * **(6.6;43.0%)**
10–14	552.5 (334.0;826.9)	552.8 (340.6;833.7)	**7.4%** **(−1.4;15.9%)**	340.7 (202.1;511.0)	341.6 (202.1;518.0)	6.6% (−2.9;16.9%)
15–19	843.3 (514.8;1263.2)	852.9 (517.8;1277.1)	7.4% * **(1.4;13.5%)**	557.8 (330.2;858.5)	558.3 (331.1;858.3)	1.8% (−3.8;7.8%)
**South-East Asia Region-TTH**						
5–9	23.3 (9.6;46.5)	23.4 (9.6;46.3)	18.1% * **(10.7;25.9%)**	21.6 (8.8;43.3)	21.6 (8.6;43.5)	21.9% * **(13.0;31.8%)**
10–14	79.6 (39.1;144.2)	79.9 (39.9;144.9)	7.6% * **(2.8;12.9%)**	71.1 (32.9;131.0)	71.3 (32.7;131.7)	6.7% (−0.2;13.4%)
15–19	113.5 (60.2;189.1)	114.5 (60.4;191.0)	7.2% * **(2.6;12.3%)**	90.6 (46.2;156.0)	90.8 (45.6;155.9)	1.9% (−2.5;6.5%)
**Western Pacific Region-Migraine**						
5–9	52.5 (22.9;98.6)	52.6 (22.7;97.3)	−2.1% (−13.6;10.7%)	33.0 (14.4;61.3)	32.2 (15.0;62.0)	−0.7% (−17.1;17.7%)
10–14	300.3 (179.0;458.3)	304.0 (183.2;465.6)	0.6% (−4.4;6.1%)	189.6 (111.8;291.3)	192.9 (113.7;298.3)	1.2% (−4.9;7.7%)
15–19	486.3 (291.8;744.7)	511.0 (304.7;708.1)	3.9% (−0.2;8.5%)	307.9 (185.1;467.0)	328.4 (199.7;501.7)	4.1% (−0.6;9.1%)
**Western Pacific Region-TTH**						
5–9	19.1 (7.9;38.0)	19.2 (7.8;37.5)	−1.8% (−7.7;4.3%)	16.7 (6.7;33.3)	16.8 (6.8;34.1)	−0.8% (−8.5;6.5%)
10–14	64.9 (31.4;118.4)	64.9 (31.7;117.3)	−0.5% (−4.9%4.0%)	56.1 (25.9;102.0)	56.3 (26.3;102.5)	−0.1% (−4.8;5.0%)
15–19	83.0 (43.3;138.9)	83.3 (43.4;140.0)	−0.7% (−5.4;4.3%)	68.9 (34.7;118.5)	69.5 (118.6)	−1.7% (−6.2;3.2%)

**Note**. Percentages in bold and with * indicate significant variation.

## Data Availability

Data used for this publication are available on the website of the Institute for Health Metrics and Evaluation (available at http://ghdx.healthdata.org/gbd-results-tool) and can be browsed or downloaded with a free access.
